# Anti-inflammatory diet, physical activity, and risk of all-cause mortality in older adults: a prospective cohort study

**DOI:** 10.3389/fimmu.2026.1871177

**Published:** 2026-08-19

**Authors:** Minghui Sun, Tian Li, Ling Cong, Xinyan Ma, Mengdi Hou, Hao Ai, Zhiyun Wu, Chao Huang

**Affiliations:** 1 The Third Affiliated Hospital of Jinzhou Medical University, Jinzhou, China; 2 Liaoning Key Laboratory of Precision Medical Research on Major Chronic Disease, Shenyang, China; 3 Shengjing Hospital of China Medical University, Shenyang, China; 4 Liaoning Provincial Key Laboratory of Follicular Development and Reproductive Health, Jinzhou, China

**Keywords:** anti-inflammatory diet, dietary pattern, mortality, older adults, physical activity

## Abstract

**Background:**

Anti-inflammatory dietary patterns are associated with a lower risk of several chronic diseases, but their association with all-cause mortality in Chinese older adults is unknown. In this first study of a Chinese population, we investigated this association and its joint effects with physical activity, to inform strategies for healthy longevity.

**Methods:**

This analysis included 6,468 participants aged ≥65 years from the Chinese Longitudinal Healthy Longevity Survey. Anti-inflammatory diet was assessed using the anti-inflammatory diet index (AIDI), which was derived from data collected via a simplified food frequency questionnaire. Physical activity was assessed by a self-reported questionnaire. We used Cox proportional hazards regression models to estimate hazard ratios (HRs) and 95% confidence intervals (CIs).

**Results:**

During a median follow-up of 7 years, 4,117 deaths occurred. After multivariable adjustment, compared with participants in the lowest tertile of the AIDI, those in the highest tertile had a lower risk of all-cause mortality (HR = 0.85; 95%CI: 0.77-0.94). Each one standard deviation increase in the AIDI was associated with a 8% lower risk of mortality. Joint analyses further showed that a high AIDI combined with regular exercise, or with high leisure activity, was associated with a significantly lower mortality risk.

**Conclusions:**

Adherence to an anti-inflammatory diet was associated with reduced mortality risk in Chinese older adults. The findings underscore the value of integrating both dietary and physical activity strategies to promote healthy longevity.

## Introduction

The global population is ageing at an accelerating pace, and the health and longevity of older adults have emerged as a core public health priority for society at large (1, 2). As population life expectancy increases, physiological functions decline in older people, and the immune system tends to enter a state of low-grade, persistent activation (3, 4). Over time, the accumulation of such symptoms increases the risk of several chronic conditions (e.g., hypertension, diabetes, and malignancies), which in turn elevates all-cause mortality (5–7). As an important ultimate indicator of health outcomes in older people, all-cause mortality warrants investigation and intervention of its determinants, which carry substantial clinical and public health implications. Diet, as a modifiable lifestyle factor, has attracted increasing attention.

By combining foods rich in anti-inflammatory components, an anti-inflammatory diet reduces excessive free radicals, improves gut microecology, and regulates fatty acid balance, thereby alleviating inflammaging in older people (8, 9). Previous research have shown an inverse association between an anti-inflammatory diet and all-cause mortality in the Swedish population (10). Another study of 18,795 US adults reported that higher intake of anti-inflammatory foods was correlated with lower all-cause mortality (11). However, these studies have focused on Western populations, and data on these associations in Eastern populations remain scarce.

Considerable evidence has examined the association between physical activity and mortality (12, 13). Physical activity may reduce the risk of mortality, at least in part, by alleviating oxidative stress and systemic inflammation (14, 15). Nevertheless, the joint effect of an anti-inflammatory diet and physical activity on mortality risk in older adults remains unclear.

Using data from the Chinese Longitudinal Healthy Longevity Survey (CLHLS), we investigated the association between an anti-inflammatory diet and all-cause mortality in Chinese older adults and further examined the joint effects of diet and physical activity. These findings provide evidence for developing modifiable lifestyle interventions to promote longevity in older populations.

## Methods

### Study design and participants

Data used in this study were derived from the CLHLS. The CLHLS is a nationally representative longitudinal cohort study that comprehensively investigates demographic characteristics, lifestyle behaviors, disease history, mental status and other relevant dimensions among Chinese older adults aged 65 years and above, providing evidence for healthy aging in the Chinese population (16–18). We used baseline data of participants collected in 2011–2012 and followed them up until the occurrence of an outcome event or the end of follow-up in 2018. All participants provided written informed consent prior to enrollment. This study was approved by the Biomedical Ethics Committee of Peking University (IRB00001052–13074).

**Figure 1:**
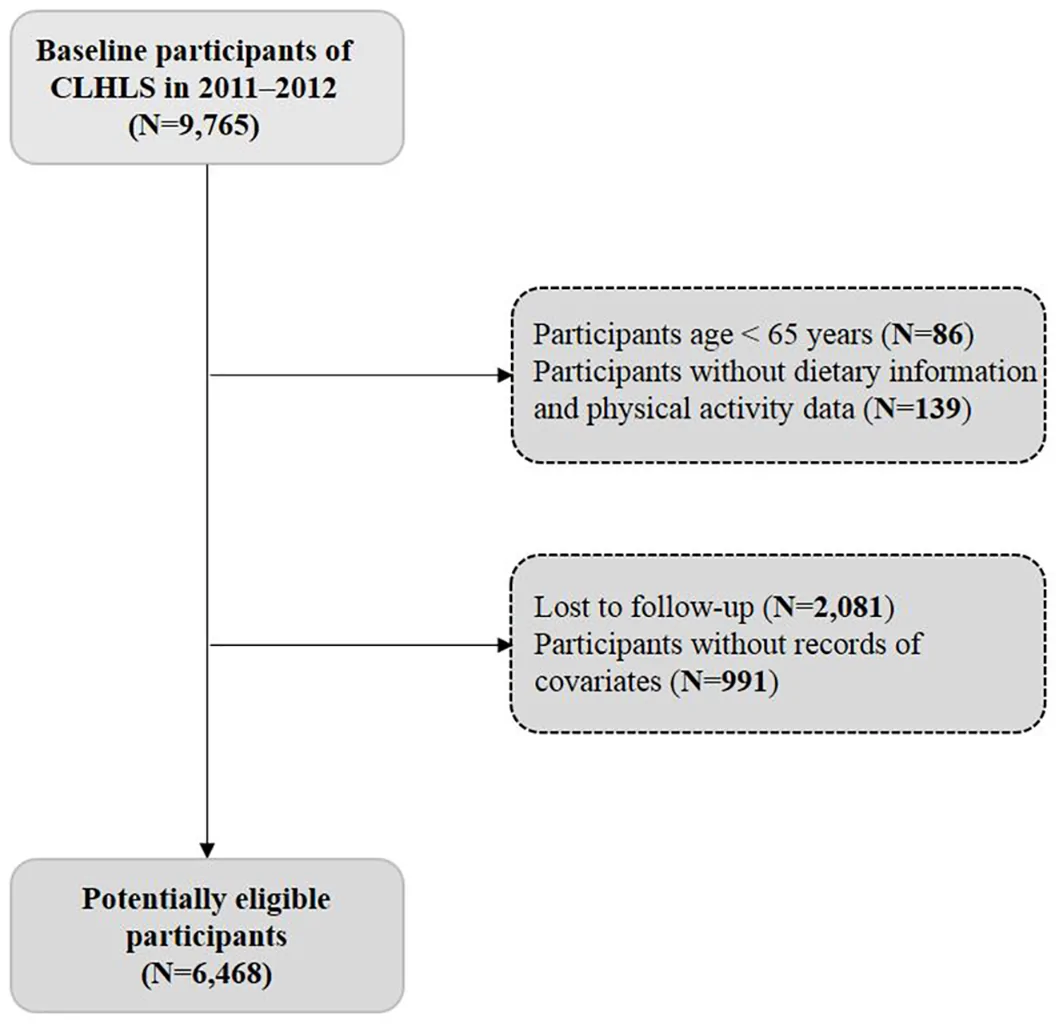
Flow chart of the study participant selection process.

Among the 9,765 participants at baseline, we excluded those aged under 65 years (*N* = 86), those with missing baseline data on diet or physical activity (*N* = 139), those lost to follow-up (*N* = 2,081), and those with missing covariate data (*N* = 991). The final analytic sample comprised 6,468 older adults (**Figure 1**).

### Assessment of anti-inflammatory diet index

We assessed dietary intake using a simplified food frequency questionnaire (FFQ) that has been extensively validated in previous studies (19–21). The questionnaire covers a range of dietary items, encompassing vegetables, fruits, nuts, soy products, dairy foods, medicinal plants, tea, and other foods group. Dietary consumption frequency was categorized into five levels: daily or almost daily, weekly, monthly, occasionally, and rarely or never.

We adopted the AIDI to assess anti-inflammatory dietary patterns among participants in the present study. Referring to existing evidence (22), the index established in our analysis consisted of ten dietary groups, including mushroom or algae, soy foods, tea, garlic, medicinal plants, fish, eggs, vitamins (a/c/e) supplement/calcium tablet, nuts, and milk or dairy. Participants received scores from 4 to 0 according to descending consumption frequency (4 = daily or almost daily, 0 = rarely or never). We then calculated the total score (0-40) for each participant, with higher scores indicating greater adherence to an anti-inflammatory diet. Detailed scoring criteria are presented in **Supplementary Table 1**.

### Assessment of physical activities

A self-reported questionnaire assessed baseline physical activities, including physical labour, regular exercise, and leisure activities (23, 24). Participants were asked whether they regularly engaged in physical labour (yes/no) and regular exercise (yes/no). Leisure activities were evaluated based on six items: housework, gardening, outdoor activities, raising livestock or pets, participating in social activities, and playing cards or mahjong. Responses for each leisure activity component were then summed, yielding a total score ranging from 6 to 30, with higher scores indicating greater participation in leisure activities. Based on the median score, participants were divided into low leisure activity and high leisure activity groups (23).

### Assessment of mortality

Baseline data were collected in 2011–2012. Mortality status was ascertained through two follow-up surveys conducted in 2014 and 2018. Follow-up time was calculated from the baseline survey in 2011–2012 until the occurrence of all-cause death or the end of follow-up in 2018. All-cause mortality was defined as the primary outcome.

### Covariates

A comprehensive set of covariates (demographic characteristics, mental health, and lifestyle factors) was collected using validated questionnaires administered by trained interviewers. These covariates comprised age, sex (male/female), residential location (urban/township/rural), ethnicity (Han/other), body mass index (BMI), annual income (≥30,000/<30,000 yuan), physical work (yes/no), regular exercise (yes/no), alcohol consumption (yes/no), current marital status (living with a partner or not), smoking status (yes/no), and hypertension (yes/no). BMI was derived from physical measurements and calculated as weight in kilograms divided by the square of height in metres.

### Statistical analyses

Continuous variables were summarised as medians (interquartile ranges [IQR]), and group comparisons were made using the Kruskal-Wallis test. Categorical variables were reported as numbers (percentages) and analysed using the χ² test. Overall survival probability was estimated by the Kaplan–Meier method, and crude survival curves were plotted.

Cox proportional hazards regression models were used to calculate hazard ratios (HRs) and 95% confidence intervals (CIs) for the associations of anti-inflammatory diet and physical activity with mortality risk. The proportional hazards assumption was tested using Schoenfeld residuals and was satisfied. Using the lowest tertile of the anti-inflammatory diet index as the reference, we obtained the *P* value for trend by modelling the median value of each tertile as a continuous variable. Dietary pattern intake was evaluated per standard deviation (SD) increment. We selected covariates based on their relevance to the anti-inflammatory diet, prior literature, and clinical considerations: age, sex, place of residence, BMI, smoking, alcohol consumption, regular exercise, physical work, marital status, ethnicity, and hypertension. In addition, we utilized restricted cubic splines (RCS) to examine nonlinear associations between the anti-inflammatory diet and mortality. Subgroup analyses were conducted by age, gender, BMI, residence, alcohol drinking, and smoking status. *P*-values for interaction were assessed using likelihood -ratio tests.

To evaluate the jointed impacts of AIDI and physical activity on the risk of mortality, participants were categorised into four groups based on AIDI (low vs high, according to the median) and physical activity category (yes vs no). The reference group consisted of participants with low AIDI and no physical activity. The relative excess risk due to interaction (25), was calculated to assess additive interaction, and the likelihood ratio test was used to evaluate multiplicative interaction. Sensitivity analyses were also conducted. First, we made additional adjustments for confounders including participants’ baseline chronic conditions (e.g., diabetes, cardiovascular disease, respiratory disease) and general health status. Second, we compared the demographic, clinical, and lifestyle characteristics between included and excluded participants. Third, we performed multiple imputation for missing covariates. Finally, E-value analyses were conducted to assess the potential impact of residual confounding.

All statistical tests were two-sided, with *P* < 0.05 considered statistically significant. Analyses were performed using SAS version 9.4.

## Results

### Baseline characteristics of participants

Our final study included 6,468 participants with a baseline median age of 87 years (IQR 77-95); 55.7% were women. During follow-up, 4,117 participants died. Comparing baseline characteristics between survivors and non-survivors, we found lower mortality rates among those who lived with a partner, consumed alcohol, smoked, exercised regularly, engaged in physical labour, or participated in high-level leisure activities. In addition, participants of older age had higher mortality (**Table 1**).

**Table 1:** Baseline characteristics of the study population.

Characteristics	Total	Status of follow-up		*P* valve
		Surviving	Dead	
Number of participants, n	6,468	2,351	4,117	
Age (years)	87.00 (77.00, 95.00)	77.00 (72.00, 84.00)	92.00 (85.00, 98.00)	< 0.01
BMI (kg/m^2^)	20.28 (17.78, 22.96)	21.64 (19.29, 24.22)	19.50 (17.30, 22.10)	< 0.01
AIDI (scores)	14.00 (9.00, 18.00)	14.00 (10.00, 19.00)	13.00 (9.00, 18.00)	< 0.01
Gender, n (%)				0.05
Men	2,868 (44.34)	1,080 (45.94)	1,788 (43.43)	
Women	3,600 (55.66)	1,271 (54.06)	2,329 (56.57)	
Marital status, n (%)				< 0.01
Live with spouse	2,242 (34.66)	1,264 (53.76)	978 (23.76)	
Live without spouse	4,226 (65.34)	1,087 (46.24)	3,139 (76.24)	
Race, n (%)				0.68
Han	6,050 (93.54)	2,203 (93.70)	3,847 (93.44)	
Other	418 (6.46)	148 (6.30)	270 (6.56)	
Residence, n (%)				0.08
City	1,011 (15.63)	344 (14.63)	667 (16.20)	
Town	2,125 (32.85)	756 (32.16)	1,369 (33.25)	
Rural	3,332 (51.52)	1,251 (53.21)	2,081 (50.55)	
Household annual income, n (%)				< 0.01
< 30,000 yuan	4,493 (69.47)	1,682 (71.54)	2,811 (68.28)	
≥ 30,000 yuan	1,975 (30.53)	669 (28.46)	1,306 (31.72)	
Smoking status, n (%)				< 0.01
No	5,350 (82.71)	1,888 (80.31)	3,462 (84.09)	
Yes	1,118 (17.29)	463 (19.69)	655 (15.91)	
Drinking status, n (%)				< 0.01
No	5,349 (82.70)	1,862 (79.20)	3,487 (84.70)	
Yes	1,119 (17.30)	489 (20.80)	630 (15.30)	
Regular exercise, n (%)				< 0.01
No	4,307 (66.59)	1,346 (57.25)	2,961 (71.92)	
Yes	2,161 (33.41)	1,005 (42.75)	1,156 (28.08)	
Labor status, n (%)				0.11
No	951 (14.70)	324 (13.78)	627 (15.23)	
Yes	5,517 (85.30)	2,027 (86.22)	3,490 (84.77)	
Leisure activities, n (%)				< 0.01
Low leisure activities	4,662 (72.08)	1,233 (52.45)	3,429 (83.29)	
High leisure activities	1,806 (27.92)	1,118 (47.55)	688 (16.71)	
Hypertension, n (%)				0.07
No	3,065 (47.39)	1,079 (45.90)	1,986 (48.24)	
Yes	3,403 (52.61)	1,272 (54.10)	2,131 (51.76)	

AIDI, anti-inflammatory diet index; BMI, body mass index.

Values are numbers (percentages) for categorical variables and median (interquartile range) for continuous variables.

### Anti-inflammatory diet and mortality

**Table 2** shows the association between the AIDI and mortality. After full adjustment for confounding factors, participants in the highest tertile of the AIDI had a lower risk of all-cause mortality than those in the lowest tertile (HR: 0.85, 95% CI: 0.77 to 0.94) (**Supplementary Figure 1**). Each SD increase in AIDI was correlated with an 8% reduction in mortality risk. RCS analysis indicated a linear relationship between AIDI and mortality (**Supplementary Figure 2**).

**Table 2:** Association between anti-inflammatory diet and all-cause mortality.

Variables	Anti-inflammatory diet index			*P* for trend *	Continuous **
	Tertile 1	Tertile 2	Tertile 3		
AIDI					
* n/N*	1,423/2,103	1,217/1,862	1,477/2,503		4,117/6,468
Model 1 HR (95 % CI)	1.00 (Reference)	0.96 (0.89, 1.03)	0.80 (0.75, 0.87)	< 0.01	0.89 (0.87, 0.92)
Model 2 HR (95 % CI)	1.00 (Reference)	0.99 (0.90, 1.10)	0.86 (0.78, 0.94)	< 0.01	0.93 (0.89, 0.96)
Model 3 HR (95 % CI)	1.00 (Reference)	0.98 (0.89, 1.08)	0.85 (0.77, 0.94)	< 0.01	0.92 (0.89, 0.96)

AIDI, anti-inflammatory diet index; CI, confidence interval; HR, hazard ratio; SD, standard deviation.

Model 1: Crude model;

Model 2: Adjusted for age (years), gender (men, women), and BMI (kg/m^2^);

Model 3: Further adjusted for annual income level (≥30,000, <30,000 yuan), ethnicity (Han or others), regular exercise (yes, no), labor status (yes, no), marital status (live with spouse, live without spouse), residence (city, town, or rural), smoking status (yes, no), drinking status (yes, no), and hypertension (yes, no).

*P value for linear trend calculated from category median values.

**Continuous intakes were calculated by per SD increase.

**Supplementary Table 2** shows the associations of individual food components of the AIDI with mortality. Consumption of mushrooms, legumes, or garlic at least once per month was associated with lower mortality compared with no consumption. Similarly, nut intake was inversely correlated with mortality (versus none), and daily tea intake was also inversely correlated with mortality (versus none).

The inverse association between mortality and anti-inflammatory diet persisted across subgroups of participants aged ≥80 years, women, rural residents, normal BMI, and non-smoking non-drinkers. We also observed a significant interaction between BMI and the anti-inflammatory diet (**Supplementary Table 3**).

### Physical activity and mortality

The associations of physical activity with mortality are shown in **Table 3**. Compared with participants who did no physical labour, those who engaged in physical labour had a lower mortality risk (HR: 0.88, 95% CI: 0.79 to 0.98). Compared with those who exercised irregularly, participants who exercised regularly had a significantly lower mortality risk (HR: 0.74, 95% CI: 0.68 to 0.80). Compared with low leisure activities, participation in high leisure activities were correlated with a lower mortality risk (HR: 0.61, 95% CI: 0.55 to 0.67).

**Table 3:** Association between physical activities and all-cause mortality.

Variables	*n/N*	Model 1 HR (95%CI)	Model 2 HR (95%CI)	Model 3 HR (95%CI)
Physical work				
No	627/951	1.00 (Reference)	1.00 (Reference)	1.00 (Reference)
Yes	3,490/5,517	0.90 (0.83, 0.98)	0.88 (0.79, 0.98)	0.88 (0.79, 0.98)
Regular exercise				
No	2,961/4,307	1.00 (Reference)	1.00 (Reference)	1.00 (Reference)
Yes	1,156/2,161	0.64 (0.60, 0.68)	0.76 (0.70, 0.82)	0.74 (0.68, 0.80)
Leisure activities				
Low leisure activities	3,429/4,662	1.00 (Reference)	1.00 (Reference)	1.00 (Reference)
High leisure activities	688/1,806	0.35 (0.32, 0.38)	0.56 (0.51, 0.62)	0.61 (0.55, 0.67)

CI, confidence interval; HR, hazard ratio.

Model 1: Crude model;

Model 2: Adjusted for age (years), gender (men, women), and BMI (kg/m^2^);

Model 3: Further adjusted for annual income level (≥30,000, <30,000 yuan), ethnicity (Han or others), regular exercise (yes, no), labor status (yes, no), marital status (live with spouse, live without spouse), residence (city, town, or rural), smoking status (yes, no), drinking status (yes, no), and hypertension (yes, no).

### Combined associations of AIDI and physical activities with mortality

**Figure 2:**
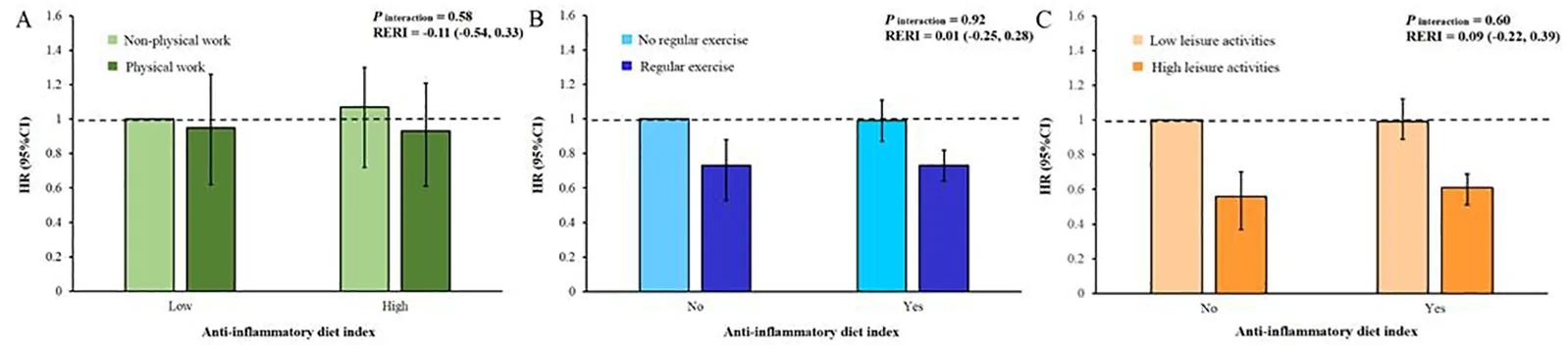
Combined effects of anti-inflammatory diet and physical work **(A)**, regular exercise **(B)**, and leisure activities **(C)**. CI, confidence interval; HR, hazard ratio; RERI, relative excess risk due to interaction. HRs and 95% CIs were calculated using Cox proportional hazards regression model adjusted for age (years), gender (men, women), BMI (kg/m^2^), annual income level (≥30,000, <30,000 yuan), ethnicity (Han or others), regular exercise (yes, no), labor status (yes, no), marital status (live with spouse, live without spouse), residence (city, town, or rural), smoking status (yes, no), drinking status (yes, no), and hypertension (yes, no).

The results of the joint analysis are shown in **Figure 2**. Compared with participants with low AIDI and no physical labour, those with high AIDI and physical labour showed no significant association with mortality (HR: 0.93, 95% CI: 0.68 to 1.28). Compared with participants with low AIDI and irregular exercise, those with high AIDI and regular exercise were associated with a reduced risk of mortality (HR: 0.73, 95% CI: 0.64 to 0.84). Compared with participants with low AIDI and low leisure activity, those with high AIDI and high leisure activity were associated with a significantly reduced risk of mortality (HR: 0.61, 95% CI: 0.53 to 0.71).

### Sensitivity analysis

Sensitivity analyses with additional adjustment for confounders including baseline chronic diseases and general health status yielded consistent directional findings relative to the main analysis (**Supplementary Table 4**). Comparisons between included and excluded participants revealed that the two groups did not differ statistically in the vast majority of characteristics (**Supplementary Table 5**). Findings from analyses after multiple imputation were consistent in direction with those of the primary analysis (**Supplementary Table 6**). E-value indicated that the association between AIDI, physical activity, and mortality was robust to unmeasured confounding (**Supplementary Table 7**).

## Discussion

This prospective study showed that adherence to an anti-inflammatory diet was associated with lower all-cause mortality in older adults. Physical activity was similarly inversely correlated with all-cause mortality. In joint analysis, the combination of high AIDI and regular exercise was associated with a 27% lower mortality risk, and high AIDI combined with high leisure activity was associated with a 39% lower mortality risk.

Current evidence on the association between an anti-inflammatory diet and mortality remains limited, with only a few studies have explored this relationship. Existing cohort studies provide preliminary support. For example, one investigation of Swedish adults found that anti-inflammatory dietary patterns were related to a reduced risk of mortality (10), while another US-based study reported an inverse association between all-cause mortality and anti-inflammatory diet (11). Our study adds evidence from the Chinese population suggesting that adherence to an anti-inflammatory diet was associated with a lower risk of all-cause mortality in older adults. Mechanistically, these protective effects may be partly explained by modulation of gut microbiota composition (26, 27), mitigation of chronic inflammation (28), and reduction of oxidative stress (29). Such biological processes may collectively delay senescence, slow the progression of age-related chronic diseases, and ultimately contribute to lower long-term mortality.

Interestingly, we observed heterogeneity in the association between all-cause mortality and anti-inflammatory diet according to body weight status. Among normal-weight older adults, adherence to an anti-inflammatory diet was inversely correlated with all-cause mortality, but no statistically significant association was found in those who were underweight or obese. This finding warrants further mechanistic investigation. We also observed an interaction between BMI and the anti-inflammatory diet on mortality. One potential explanation was that individuals with normal BMI have lower levels of chronic low-grade inflammation and may therefore derive greater survival benefits from an anti-inflammatory dietary pattern (30, 31). In contrast, excess adiposity in overweight or obesity may induce chronic systemic inflammation that outweighs the relatively modest anti-inflammatory effects of diet, thereby attenuating or masking the protective association (31, 32). Furthermore, obesity-related metabolic disturbances (e.g., insulin resistance, dyslipidaemia, and oxidative stress) may interfere with the pathways through which anti-inflammatory nutrients benefit health (33, 34). These results suggest that the survival benefits of an anti-inflammatory diet may be modified by adiposity, with normal-weight individuals deriving greater benefit from such dietary strategies.

In joint analyses, high AIDI and regular exercise or leisure activity significantly reduced all-cause mortality, but the protective impact of the diet was not significant among participants doing physical work. This discrepancy may be explained by differences in activity characteristics. Moderate, rhythmic leisure exercise reduces systemic low-grade inflammation, oxidative stress, and metabolic dysfunction (35, 36). Paired with anti-inflammatory diets rich in polyphenols and antioxidants, moderate leisure exercise may synergistically slow inflammaging, prevent chronic diseases, and lower mortality. Leisure activity also promotes better sleep, reduces stress, and encourages healthier lifestyles, which may further enhance the protective effects of an anti-inflammatory diet (37, 38). By contrast, prolonged high-intensity physical labor induces chronic fatigue, musculoskeletal injury, and sustained physiological stress (39). It triggers oxidative overload, chronic inflammation, and HPA axis dysfunction, thereby offsetting the diet’s anti-inflammatory and antioxidant benefits (40, 41). Additionally, manual workers often have irregular diets, unhealthy behaviours, and long-term exposure to occupational hazards, which may further attenuate the survival benefits of an anti-inflammatory diet (42, 43). Thus, the benefits of an anti-inflammatory diet are activity-dependent, not universal. Our findings call for personalised lifestyle strategies based on activity type, instead of one-size-fits-all advice on physical activity and anti-inflammatory diets.

This is the first study to investigate the association between an anti-inflammatory diet and all-cause mortality in older Chinese adults, and to examine its combined effects with physical activity. Our findings provide evidence on dietary patterns and lifestyle modifications to promote healthy ageing in older populations.

Several limitations of this study warrant acknowledgement. First, although this study adopted a prospective design, causal inferences cannot be drawn. Furthermore, reverse causality between exposure and outcome cannot be ruled out. Second, the AIDI adopted in the present study mainly captures anti-inflammatory dietary components, while several typical pro-inflammatory dietary factors were not incorporated into the index, such as processed meat, ultra-processed foods, saturated fat, added sugar and glycemic load. In addition, the food frequency questionnaire from the CLHLS only recorded the consumption frequency of each food item rather than precise quantitative intake, which may introduce mild exposure misclassification and inevitably bring certain measurement bias to our dietary assessment. Future studies are warranted to perform broader external validation of the AIDI. Additionally, researchers should adopt validated semi-quantitative FFQs to achieve precise assessment of participants’ dietary consumption levels. Third, physical activity was assessed using a single self-reported questionnaire, which may have introduced recall bias. Internationally standardised physical activity assessment scales should be used in future studies. Finally, although a wide range of confounders were accounted for in our analyses, residual and unmeasured confounding (such as functional disability, nutritional status, and access to healthcare) may still bias the observed associations. Nevertheless, the E-value analysis indicated that only substantial residual confounding could fully explain the observed associations.

## Conclusions

Our findings indicate that adherence to an anti-inflammatory diet was associated with a lower risk of all-cause mortality in older adults. Similarly, physical activity was inversely correlated with mortality risk. In combined analyses, a high anti-inflammatory diet plus regular exercise or leisure activity was correlated with a substantial reduction in all-cause mortality risk. These observations support the beneficial roles of dietary patterns and physical activity in promoting healthy longevity among older adults, with implications for future health promotion strategies in ageing populations.

## Data Availability

The raw data supporting the conclusions of this article will be made available by the authors, without undue reservation.
