# Supplementary material for: Anti-inflammatory diet, physical activity, and risk of all-cause mortality in older adults: a prospective cohort study
Source: Front Immunol. 2026 Aug 19;17:1871177. doi: 10.3389/fimmu.2026.1871177 (PMC13534123; doi:10.3389/fimmu.2026.1871177)
Supplement: Supplementary file 1 [file SupplementaryFile1.docx]

**Supplementary Table 1**. Calculation of anti-inflammatory diet index.

| **Components** | **4 points** | **3 points** | **2 points** | **1 points** | **0 points** |
| --- | --- | --- | --- | --- | --- |
| Mushroom or algae | 1 | 2 | 3 | 4 | 5 |
| Medicinal plants | 1 | 2 | 3 | 4 | 5 |
| Legumes | 1 | 2 | 3 | 4 | 5 |
| Nuts | 1 | 2 | 3 | 4 | 5 |
| Garlic | 1 | 2 | 3 | 4 | 5 |
| Fish | 1 | 2 | 3 | 4 | 5 |
| Eggs | 1 | 2 | 3 | 4 | 5 |
| Milk and dairy | 1 | 2 | 3 | 4 | 5 |
| Vitamins (a/c/e) supplement/  calcium tablet | 1 | 2 | 3 | 4 | 5 |
| Tea | 1 | 2 | 3 | 4 | 5 |
| **Total score** | **40 points** | | | | |

In the categories of food groups, the terms 1, 2, 3, 4, and 5 correspond to “almost every day,” “not every day but at least once a week,” “not every week but at least once a month,” “not every month but occasionally,” and “rarely or never”.

**Supplementary Table 2**. Association between individual components of anti-inflammatory diet and all-cause mortality.

| **Characteristic** | *****N* _total_ (%)**** |  | |
| --- | --- | --- | --- |
|  |  | ****HR (95 % CI)**** | ***P* _value_** |
| **Mushroom or algae** |  |  |  |
| Rarely or never | 3,026 (46.78) | 1.00 (Reference) |  |
| Not every month but occasionally | 1,528 (23.63) | 0.89 (0.80, 0.98) | 0.02 |
| Not every week but at least once a month | 944 (14.59) | 0.93 (0.83, 1.05) | 0.25 |
| Not every day but at least once a week | 857 (13.25) | 0.84 (0.74, 0.95) | < 0.01 |
| Almost every day | 113 (1.75) | 0.40 (0.21, 0.77) | 0.22 |
| **Medicinal plants** |  |  |  |
| Rarely or never | 5,438 (84.08) | 1.00 (Reference) |  |
| Not every month but occasionally | 524 (8.10) | 0.91 (0.80, 1.04) | 0.16 |
| Not every week but at least once a month | 185 (2.86) | 0.96 (0.76, 1.20) | 0.69 |
| Not every day but at least once a week | 154 (2.38) | 1.14 (0.88, 1.47) | 0.32 |
| Almost every day | 167 (2.58) | 1.01 (0.81, 1.27) | 0.93 |
| **Legumes** |  |  |  |
| Rarely or never | 922 (14.25) | 1.00 (Reference) |  |
| Not every month but occasionally | 858 (13.27) | 0.93 (0.81, 1.08) | 0.34 |
| Not every week but at least once a month | 1,171 (18.10) | 0.90 (0.78, 1.03) | 0.14 |
| Not every day but at least once a week | 2,654 (41.04) | 0.85 (0.76, 0.96) | < 0.01 |
| Almost every day | 863 (13.34) | 0.91 (0.78, 1.05) | 0.18 |
| **Nuts** |  |  |  |
| Rarely or never | 4,159 (64.30) | 1.00 (Reference) |  |
| Not every month but occasionally | 1,022 (15.80) | 0.86 (0.77, 0.96) | < 0.01 |
| Not every week but at least once a month | 532 (8.23) | 0.78 (0.66, 0.91) | < 0.01 |
| Not every day but at least once a week | 571 (8.83) | 0.78 (0.67, 0.90) | < 0.01 |
| Almost every day | 184 (2.84) | 0.67 (0.52, 0.86) | < 0.01 |
| **Fish** |  |  |  |
| Rarely or never | 1,437 (22.22) | 1.00 (Reference) |  |
| Not every month but occasionally | 929 (14.36) | 0.95 (0.84, 1.08) | 0.43 |
| Not every week but at least once a month | 1,287 (19.90) | 0.85 (0.76, 0.96) | < 0.01 |
| Not every day but at least once a week | 2,336 (36.12) | 0.89 (0.80, 0.98) | 0.02 |
| Almost every day | 479 (7.42) | 0.81 (0.68, 0.96) | 0.01 |
| **Eggs** |  |  |  |
| Rarely or never | 774 (11.97) | 1.00 (Reference) |  |
| Not every month but occasionally | 523 (8.09) | 0.87 (0.73, 1.04) | 0.13 |
| Not every week but at least once a month | 746 (11.53) | 0.86 (0.73, 1.01) | 0.06 |
| Not every day but at least once a week | 2,367 (36.60) | 0.90 (0.79, 1.02) | 0.11 |
| Almost every day | 2,058 (31.82) | 0.94 (0.82, 1.08) | 0.35 |
| **Milk and dairy** |  |  |  |
| Rarely or never | 3,078 (47.59) | 1.00 (Reference) |  |
| Not every month but occasionally | 771 (11.92) | 1.04 (0.91, 1.18) | 0.58 |
| Not every week but at least once a month | 548 (8.47) | 1.02 (0.88, 1.18) | 0.82 |
| Not every day but at least once a week | 901 (13.93) | 1.01 (0.90, 1.13) | 0.91 |
| Almost every day | 1,170 (18.09) | 0.99 (0.90, 1.11) | 0.95 |
| **Garlic** |  |  |  |
| Rarely or never | 2,198 (33.98) | 1.00 (Reference) |  |
| Not every month but occasionally | 973 (15.04) | 0.89 (0.79, 1.01) | 0.06 |
| Not every week but at least once a month | 715 (11.05) | 0.95 (0.83, 1.09) | 0.50 |
| Not every day but at least once a week | 1,399 (21.64) | 0.84 (0.76, 0.94) | < 0.01 |
| Almost every day | 1,183 (18.29) | 1.00 (0.89, 1.12) | 0.99 |
| **Vitamins (a/c/e) supplement/calcium tablet** |  |  |  |
| Rarely or never | 4,912 (75.94) | 1.00 (Reference) |  |
| Not every month but occasionally | 606 (9.37) | 0.93 (0.82, 1.06) | 0.29 |
| Not every week but at least once a month | 193 (2.98) | 0.85 (0.67, 1.06) | 0.15 |
| Not every day but at least once a week | 278 (4.30) | 0.99 (0.82, 1.18) | 0.89 |
| Almost every day | 479 (7.41) | 0.99 (0.85, 1.14) | 0.80 |
| **Tea** |  |  |  |
| Rarely or never | 3,937 (60.87) | 1.00 (Reference) |  |
| Not every month but occasionally | 390 (6.03) | 0.93 (0.78, 1.10) | 0.40 |
| Not every week but at least once a month | 183 (2.83) | 1.02 (0.80, 1.28) | 0.90 |
| Not every day but at least once a week | 442 (6.83) | 1.01 (0.87, 1.17) | 0.94 |
| Almost every day | 1,516 (23.44) | 0.86 (0.78, 0.95) | < 0.01 |

CI, confidence interval; HR, hazard ratio.

HRs and 95 % CIs were adjusted for age, gender, BMI, annual income level, ethnicity, regular exercise, labor status, marital status, residence, smoking status, drinking status, and hypertension.

**Supplementary Table 3.** Subgroup analyses of anti-inflammatory diet and all-cause mortality.

|  | *N* _cases_/*N* _total_ | **HR (95 % CI) *** | *P* _value_ | *P* _interaction**_ |
| --- | --- | --- | --- | --- |
| Age (years) |  |  |  | 0.64 |
| 65-79 | 533/1,963 | 0.88 **(**0.68, 1.13**)** | 0.31 |  |
| ≥80 | 3,584/4,505 | 0.88 **(**0.79, 0.93**)** | 0.01 |  |
| Gender |  |  |  | 0.52 |
| Male | 1,788/2,868 | 0.92 (0.79, 1.07) | 0.27 |  |
| Female | 2,329/3,600 | 0.82 (0.71, 0.93) | < 0.01 |  |
| Residence |  |  |  | 0.13 |
| City | 667/1,011 | 0.80 (0.61, 1.05) | 0.11 |  |
| Town | 1,369/2,125 | 0.98 (0.84, 1.15) | 0.81 |  |
| Rural | 2,081/3,332 | 0.82 (0.71, 0.94) | < 0.01 |  |
| BMI (kg/m^2^) |  |  |  | < 0.01 |
| <18.5 | 2,378/3,331 | 0.91 (0.81, 1.01) | 0.07 |  |
| 18.5-23.9 | 1,356/2,296 | 0.86 (0.75, 0.99) | 0.04 |  |
| ≥ 24 | 383/841 | 0.89 (0.67, 1.17) | 0.40 |  |
| Smoking status |  |  |  | 0.30 |
| No | 3,462/5,350 | 0.83 (0.75, 0.93) | < 0.01 |  |
| Yes | 655/1,118 | 0.96 (0.76, 1.22) | 0.74 |  |
| Drinking status |  |  |  | 0.80 |
| No | 3,487/5,349 | 0.85 (0.76, 0.95) | < 0.01 |  |
| Yes | 630/1,119 | 0.88 (0.68, 1.14) | 0.32 |  |

BMI, body mass index; CI, confidence interval; HR, hazard ratio.

HRs and 95 % CIs were adjusted for age, gender, BMI, annual income level, ethnicity, exercise status, labor status, marital status, residence, smoking status, drinking status, and hypertension.

**Supplementary Table 4.** Sensitivity analyses: Association between anti-inflammatory diet and all-cause mortality with additional adjustment for chronic diseases and general health status.

| **Variables** | Anti-inflammatory diet index | | | *P* for trend * | Continuous ** |
| --- | --- | --- | --- | --- | --- |
|  | Tertile 1 | Tertile 2 | Tertile 3 |  |  |
| **AIDI** |  |  |  |  |  |
| *n/N* | 1,423/2,103 | 1,217/1,862 | 1,477/2,503 |  | 4,117/6,468 |
| Model 1 **HR (95 % CI)** | 1.00 (Reference) | 0.96 (0.89, 1.03) | 0.80 (0.75, 0.87) | < 0.01 | 0.89 (0.87, 0.92) |
| Model 2 **HR (95 % CI)** | 1.00 (Reference) | 0.99 (0.90, 1.10) | 0.86 (0.78, 0.94) | < 0.01 | 0.93 (0.89, 0.96) |
| Model 3 **HR (95 % CI)** | 1.00 (Reference) | 1.00 (0.89, 1.12) | 0.89 (0.80, 0.99) | 0.04 | 0.94 (0.89, 0.98) |

AIDI, anti-inflammatory diet index; CI, confidence interval; HR, hazard ratio; SD, standard deviation.

Model 1: Crude model;

Model 2: Adjusted for age, gender, and BMI;

Model 3: Further adjusted for annual income level, ethnicity, regular exercise, labor status, marital status, residence, smoking status, drinking status, hypertension, diabetes mellitus, cardiovascular diseases, stroke, respiratory diseases, and self-reported health status.

* *P* value for linear trend calculated from category median values.

** Continuous intakes were calculated by per SD increase

**Supplementary Table 5.** Characteristics of participants in the sensitivity analysis.

| **Characteristics** | **All participants in the main analysis** | **Participants excluded from the analysis** | ***P*-value** |
| --- | --- | --- | --- |
| Number of participants, n | 6,468 | 3,006 |  |
| Age (years) | 87.00 (77.00, 95.00) | 84.00 (75.00, 93.00) | 0.11 |
| BMI (kg/m^2^) | 20.28 (17.78, 22.96) | 20.81 (18.60, 23.44) | < 0.01 |
| Gender, n (%) |  |  | 0.14 |
| Men | 2,868 (44.34) | 1,382 (45.97) |  |
| Women | 3,600 (55.56) | 1,624 (54.03) |  |
| Marital status, n (%) |  |  | < 0.01 |
| Live with spouse | 2,242 (34.66) | 1,167 (40.52) |  |
| Live without spouse | 4,226 (65.34) | 1,713 (59.48) |  |
| Race, n (%) |  |  | 0.22 |
| Han | 6,050 (93.54) | 2,091 (95.70) |  |
| Other | 418 (6.46) | 94 (4.30) |  |
| Residence, n (%) |  |  | < 0.01 |
| City | 1,011 (15.63) | 695 (23.12) |  |
| Town | 2,125 (32.85) | 689 (22.92) |  |
| Rural | 3,332 (51.52) | 1,622 (53.96) |  |
| Household annual income, n (%) |  |  | 0.15 |
| < 30,000 yuan | 4,493 (69.47) | 2,044 (68.00) |  |
| ≥ 30,000 yuan | 1,975 (30.53) | 962 (32.00) |  |
| Smoking status, n (%) |  |  | 0.41 |
| No | 5,350 (82.71) | 2,406 (80.98) |  |
| Yes | 1,118 (17.29) | 565 (19.02) |  |
| Drinking status, n (%) |  |  | 0.12 |
| No | 5,349 (82.70) | 2,455 (83.99) |  |
| Yes | 1,119 (17.30) | 468 (16.01) |  |
| Regular exercise, n (%) |  |  | 0.67 |
| No | 4,307 (66.59) | 1,950 (67.03) |  |
| Yes | 2,161 (33.41) | 959 (32.97) |  |
| Hypertension, n (%) |  |  | < 0.01 |
| No | 3,065 (47.39) | 1,326 (44.11) |  |
| Yes | 3,403 (52.61) | 1,680 (55.89) |  |

BMI, body mass index.

Number was shown as median (interquartile range) unless stated otherwise. Two-sided *P*<0.05 was considered statistically significant.

**Supplementary Table 6.** Sensitivity analyses: Association between anti-inflammatory diet and all-cause mortality after multiple imputation of missing covariate data.

| **Variables** | Anti-inflammatory diet index | | | *P* for trend * | Continuous ** |
| --- | --- | --- | --- | --- | --- |
|  | Tertile 1 | Tertile 2 | Tertile 3 |  |  |
| **AIDI** |  |  |  |  |  |
| *n/N* | 1,626/2,461 | 1,386/2,159 | 1,608/2,791 |  | 4,620/7,411 |
| Model 1 **HR (95 % CI)** | 1.00 (Reference) | 0.97 (0.90, 1.04) | 0.81 (0.75, 0.86) | < 0.01 | 0.89 (0.87, 0.92) |
| Model 2 **HR (95 % CI)** | 1.00 (Reference) | 0.99 (0.90, 1.10) | 0.86 (0.79, 0.94) | < 0.01 | 0.93 (0.89, 0.96) |
| Model 3 **HR (95 % CI)** | 1.00 (Reference) | 0.98 (0.89, 1.08) | 0.85 (0.77, 0.94) | < 0.01 | 0.92 (0.89, 0.96) |

AIDI, anti-inflammatory diet index; CI, confidence interval; HR, hazard ratio; SD, standard deviation.

Model 1: Crude model;

Model 2: Adjusted for age, gender, and BMI;

Model 3: Further adjusted for annual income level, ethnicity, regular exercise, labor status, marital status, residence, smoking status, drinking status, and hypertension.

* *P* value for linear trend calculated from category median values.

** Continuous intakes were calculated by per SD increase

**Supplementary Table 7.** Sensitivity analyses: E-values for the association of anti-inflammatory diet and physical activity with all-cause mortality.

| **Outcomes** | **Exposure** | **E-values* for HR (and for CI)** |
| --- | --- | --- |
| All-cause mortality | AIDI _T3 vs. T1_ | 1.61 (1.31) |
|  | Physical work _Yes vs. No_ | 1.53 (1.16) |
|  | Regular exercise _Yes vs. No_ | 2.04 (1.90) |
|  | Leisure activities _High median vs. Low median_ | 2.66 (2.34) |

AIDI, anti-inflammatory diet index; CI, confidence interval; HR, hazard ratio; T, tertile.

* E-value represents the minimum strength of association needed between an unmeasured confounder and both the exposure and the outcome to fully explain away the exposure-outcome association.

**Supplementary Figure 1.** Kaplan-Meier survival curves for anti-inflammatory diet.

**Supplementary Figure 2.** Restricted cubic splines for anti-inflammatory diet and all-cause mortality.

The curve was adjusted for age, gender, BMI, annual income level, ethnicity, regular exercise, labor status, marital status, residence, smoking status, drinking status, and hypertension.
